# NF-*κ*B Inhibitor Myrislignan Induces Ferroptosis of Glioblastoma Cells via Regulating Epithelial-Mesenchymal Transformation in a Slug-Dependent Manner

**DOI:** 10.1155/2023/7098313

**Published:** 2023-01-16

**Authors:** Ying Zhou, Wenxuan Qian, Xiangming Li, Wenzhi Wei

**Affiliations:** ^1^College of Animal Science and Technology, Yangzhou University, Yangzhou, China; ^2^Medical College, Yangzhou University, Yangzhou, China

## Abstract

Glioblastoma (GBM) is the most common malignant tumor of the adult central nervous system. Aberrant regulation of cell death is an important feature of GBM, and investigating the regulatory mechanisms of cell death in GBM may provide insights into development of new therapeutic strategies. We demonstrated that myrislignan has ferroptosis-promoting activity. Myrislignan is a lignan isolated from *Myristica fragrans* Houtt and an inhibitor of NF-*κ*B signaling pathway. Ferroptosis is an iron-dependent form of programmed cell death characterized by the accumulation of intracellular lipid peroxidation products. Interestingly, ferroptosis was associated with other biological processes in tumor cells such as autophagy and necroptosis. Recently, the crosstalk between epithelial-mesenchymal transition (EMT) and ferroptosis has also been reported, but the mechanisms underlying the crosstalk have not been identified. Our results indicated that myrislignan suppressed growth of GBM through EMT-mediated ferroptosis in a Slug-dependent manner. Myrislignan inhibited the activation of NF-*κ*B signaling by blocking the phosphorylation of p65 protein and induced ferroptosis through the Slug-SLC7A11 signaling pathway in GBM cells. In addition, myrislignan suppressed the progression of GBM in xenograft mouse model. Hence, our findings contribute to the understanding of EMT-induced ferroptosis and provide targets for the development of targeted therapy against GBM.

## 1. Introduction

Glioblastoma (GBM) is the most malignant tumor of the adult central nervous system [1]. Although several strategies such as surgery, radiotherapy, and temozolomide chemotherapy have been used in the treatment of GBM, the prognosis of GBM patients remains poor [2]. Aberrant regulation of apoptosis is an important feature of GBM and one of the causes of chemotherapy resistance [3]. Therefore, investigating the mechanisms of cell death regulation in GBM may identify targets for developing new therapeutic strategies.

Ferroptosis is an iron-dependent form of programmed cell death which was first reported in 2012 [4]. It is distinct from other forms of cell death such as apoptosis, necroptosis, and pyroptosis and is characterized by the accumulation of intracellular lipid peroxidation products and lipid reactive oxygen species (ROS) generated during iron metabolism [5]. Interestingly, ferroptosis is closely associated with other biological processes in tumor cells such as autophagy and necroptosis [6, 7]. Epithelial-mesenchymal transition (EMT) is a significant process involved in tumor progression in which the epithelial cells lose their junctions and polarity and acquire mesenchymal traits such as increased ability for migration and invasion [8, 9]. Although there have been reports on the crosstalk between EMT and ferroptosis in several tumors [10–12], the mechanisms underlying the crosstalk have not been identified.

In this research, we revealed an antitumor activity of myrislignan, a lignan isolated from *Myristica fragrans* Houtt [13], which was reported to affect the activity of NF-*κ*B signaling pathway and showed the capability of anti-inflammatory [14]. We investigated the inhibitory activity of myrislignan against GBM *in vivo* and *in vitro*. Meanwhile, we found that myrislignan inhibited the growth of GBM through EMT-mediated ferroptosis. In summary, our study demonstrated that myrislignan is a potential drug for suppressing GBM progression and revealed the crosstalk between EMT and ferroptosis.

## 2. Materials and Methods

### 2.1. Reagents and Antibodies

Myrislignan (S3261), Z-VAD-FMK (S7023), necrosulfonamide (S8251), ferrostatin-1 (S7243), deferoxamine mesylate (S5742), and RSL3 (S8155) were purchased from Selleck (USA). 3-MA (HY-19312) and erastin (HY-15763) were purchased from MedChemExpress (USA). Dimethyl sulfoxide (DMSO) was obtained from Servicebio (G5051, China). The following antibodies were used: anti-GAPDH (60004-1-Ig, Proteintech), anti-E-cadherin (14472, Cell Signaling Technology), anti-Snail-1 (13099-1-AP, Proteintech), anti-Slug (GTX128796, GeneTex), anti-p65 (sc-8008, Santa Cruz), anti-p-p65 (sc-136548, Santa Cruz), anti-phospho-I*κ*B-*α* (2859, Cell Signaling Technology), anti-I*κ*B-*α* (CSB-RA015761A0HU, Cusabio Technology), anti-SLC7A11 (NB300-318, Novus), anti-*β*-catenin (phospho Y142, ab27798, Abcam), anti-*β*-catenin (ab32572, Abcam), anti-Cyclin-D1 (60186-1-Ig, Proteintech), anti-Bad (9292, Cell Signaling Technology), anti-Bcl2 (15017, Cell Signaling Technology), anti-Vimentin (GB111308, Servicebio), anti-Twist (25465-1-AP, Proteintech), anti-ZEB1 (21544-1-AP, Proteintech), anti-Nrf2 (M200-3, Medical and Biological Laboratories), anti-TFR1 (ab214039, Abcam), and anti-GPX4 (67763-1-Ig, Proteintech).

### 2.2. Intracranial Xenograft Model

Intracranial xenograft model was constructed using 20 nude mice (male, 5 weeks old). The mice were reared in the specific pathogen free (SPF) environment in Institute of Comparative Medicine of Yangzhou University. 20 nude mice were divided into the experimental group (U87-injected and treated with myrislignan, *n* = 8), the control group (U87-injected and treated with DMSO, *n* = 8), and the sham operation group (PBS-injected, *n* = 4). A total of 5 × 10^5^ U87 cells suspended in 3 *μ*L PBS were stereotactically implanted into the striatum (3 mm from the midline and 2 mm lateral to the bregma) of mice brain under the anesthesia with isoflurane inhalation. 7 days after the tumor injection, mice were injected intraperitoneally with DMSO or myrislignan (5 mg/kg) every 3 days over a total of 21 days. Mice were sacrificed when they showed severe neurological symptoms or obvious weight loss. All animal experiments were approved by Experimental Animal Welfare and Ethics Committee of Institute of Comparative Medicine of Yangzhou University.

### 2.3. Cell Culture

The human glioma cell lines U87 and U251 were obtained from the Type Culture Collection of the Chinese Academy of Sciences (Shanghai, China). Cells were cultured in high-glucose Dulbecco's modified Eagle's medium (DMEM) (Gibco, USA), supplemented with 10% fetal bovine serum (FBS) (Gibco, USA), penicillin (100 U/mL), and streptomycin (100 *μ*g/mL) (Beyotime, China) at 37°C in a humidified atmosphere of 5% CO_2_.

### 2.4. Plasmid Construction and Cell Transfection

Plasmids of vector, HA-Snail1, Flag-Slug, specific shRNA targeting Slug (sh-Slug), and negative control shRNA (sh-NC) were constructed by Miaoling Biology (China). Cell transfections were carried out using Lipofectamine 3000 (L3000015, Thermo Fisher Scientific) according to the manufacturer's instructions.

### 2.5. Cell Viability Assay

U87 and U251 cells were seeded in 96-well plates (5 × 10^3^ cells/well) and treated with myrislignan for different durations. Thereafter, 10 *u*L of CCK8 (Dojindo Molecular Technologies, USA) was added per well and the cells cultured for 1 h. The number of viable cells was estimated by measuring the absorbance at 450 nm wavelength using a multimode plate reader (PerkinElmer, Germany). The assays were carried out in triplicates.

### 2.6. Colony Formation Assay

U87 cells (500 cells/well) were seeded into six-well plates and cultured for three weeks. An inverted microscope was used to observe cell colony formation. At the end of the assay, cell samples were washed with PBS, fixed with 4% paraformaldehyde and stained with crystal violet. Colonies were counted using the ImageJ software.

### 2.7. Transwell Assay

U87 and U251 cells were suspended in serum-free DMEM and seeded in the upper chambers (5 × 10^4^ cells/well) of the Transwell system (Corning, USA). DMEM supplemented with 10% FBS was added to the lower chamber to induce cell migration. The Transwell systems were cultured for 24 h and then fixed in 4% paraformaldehyde and stained with 0.5% crystal violet. Following washing with running water, the cells on the upper surface of the membrane were removed using a cotton swab. The staining patterns on the membranes were captured under an inverted microscope (Olympus BX51, Japan).

### 2.8. Wound-Healing Assay

U87 and U251 cells were seeded into 6-well plates (4 × 10^5^ cells/well) and incubated for 24 h. Wounds were created by scratching the cell layer with a 100 *u*L pipette tip. The cells were then cultured in serum-free DMEM and exposed to myrislignan. The wound healing process was monitored by capturing images at 0 and 48 h under an inverted microscope (Olympus BX51, Japan), and the wound healing percentage was analyzed.

### 2.9. MDA, GSH, and Cys Assay

The lipid peroxidation levels in U87 and U251 cells were determined by analyzing the levels of MDA, GSH, and Cys. The MDA Assay Kit, GSH Assay Kit, and Cys Assay Kit were purchased from Jiancheng (Nanjing, China). Protein concentration was determined using a BCA Kit (Beyotime, China) according to the manufacturer's instructions.

### 2.10. Liable Iron Pool Assay

Labile iron pool assay was carried out using calcein acetoxymethyl ester (Corning, USA) and DFO (Selleck, USA). Cell samples were incubated in DMEM containing calcein (8 *μ*g/mL) for 30 min. DFO was then added to cause dequenching by removing iron from calcein. The fluorescence was measured at 485 nm excitation and 535 nm emission using a multimode plate reader (PerkinElmer, Germany). The change in fluorescence reflected the labile iron pool of U87 cells.

### 2.11. Lipid ROS Assay

The lipid ROS level in U87 cells was assessed using C11-BODIPY dye (#D3861, Invitrogen). Cell samples were harvested and treated with 5 *μ*M C11-BODIPY for 30 min. Later, cells were washed twice with PBS and analyzed using a FACSCalibur flow cytometer (Becton Dickinson).

### 2.12. Transmission Electron Microscopy

Transmission electron microscopy (TEM) was used to analyze the ultrastructural of mitochondria in U87 cells. Cells were harvested, fixed in 1% OsO_4_ and embedded in Spurr's resin. Ultrathin sections were stained with uranyl acetate and examined on a transmission electron microscope (Hitachi HT7700, Tokyo, Japan).

### 2.13. Dual-Luciferase Reporter Assay

The NF-*κ*B luciferase reporter plasmid was built by Miaoling Biology (Wuhan, China). The loading control Renilla luciferase reporter plasmid was purchased from Yeasen Technology (Shanghai, China). Before the luciferase test, GBM cells were cotransfected with NF-*κ*B and Renilla luciferase reporter plasmids and then treated with increasing concentrations of Myris. Cell samples were collected 48 h after exposure to Myris, and the luminescence of firefly and Renilla was determined using a dual-luciferase reporter assay kit (Yeasen Technology, China). Relative NF-*κ*B signal activity was calculated as NF-*κ*B luciferase/Renilla luciferase.

### 2.14. Western Blot Assay

Total protein was extracted from U87 and U251 cells using RIPA lysis buffer (25 mM pH 7.6 Tris-HCl, 150 mM sodium chloride, 1% NP-40, 1% sodium deoxycholate, and 0.1% sodium dodecyl sulfate) supplemented with a protease inhibitor cocktail (Roche, IN). The protein concentration was measured using a BCA Kit (Beyotime, China). Equal amounts of protein lysates were loaded and separated on SDS–polyacrylamide gels and transferred onto 0.45-mm PVDF membranes (Millipore, Germany). The membranes were blocked with 5% bovine serum albumin (BSA) for 1 hour and incubated with primary antibody at 4°C overnight. ECL detection was conducted with horseradish peroxidase- (HRP-) conjugated secondary antibody incubation and captured using a ChemiDoc Imaging System (Bio-Rad, USA).

### 2.15. Immunofluorescence Staining

Cell samples were washed with PBS for three times at room temperature, permeabilized with 0.1% Triton X-100 and blocked with 5% goat serum. Then, the samples were incubated overnight at 4°C with anti-p65 (1 : 200) and anti-p-p65 (1 : 100). The next day, cell samples were incubated with secondary antibodies (Antigen, China), and the nuclei was counterstained with diamidino-phenyl-indole (DAPI) (Antigen, China). The images were captured under an automatic microscope (Olympus BX63, Japan).

### 2.16. Immunohistochemistry (IHC)

Formalin-fixed paraffin-embedded sections of xenografts were deparaffinized with xylene and graded ethanol. For IHC, the sections were incubated with 0.3% H_2_O_2_ in ethanol for 30 min, followed by incubation with primary antibodies overnight at 4°C. The tissues were then incubated with HRP-labelled secondary antibodies and stained using 3,3′-diaminobenzidine (DAB) (Beyotime, China). Finally, the sections were mounted with neutral gum and images captured using an automatic microscope (Olympus BX63, Japan).

### 2.17. Statistical Analysis

The data were analyzed using SPSS (Version 19.0) and expressed as the mean value ± standard deviation. The student's *t*-test was applied to compare the differences between two groups, while comparisons among more than two groups were determined with one-way ANOVA. A *P* value of less than 0.05 was considered as statistical signifcance.

## 3. Results

### 3.1. Myrislignan Inhibited the EMT and Suppressed the Growth of GBM Cells

**Figure 1:**
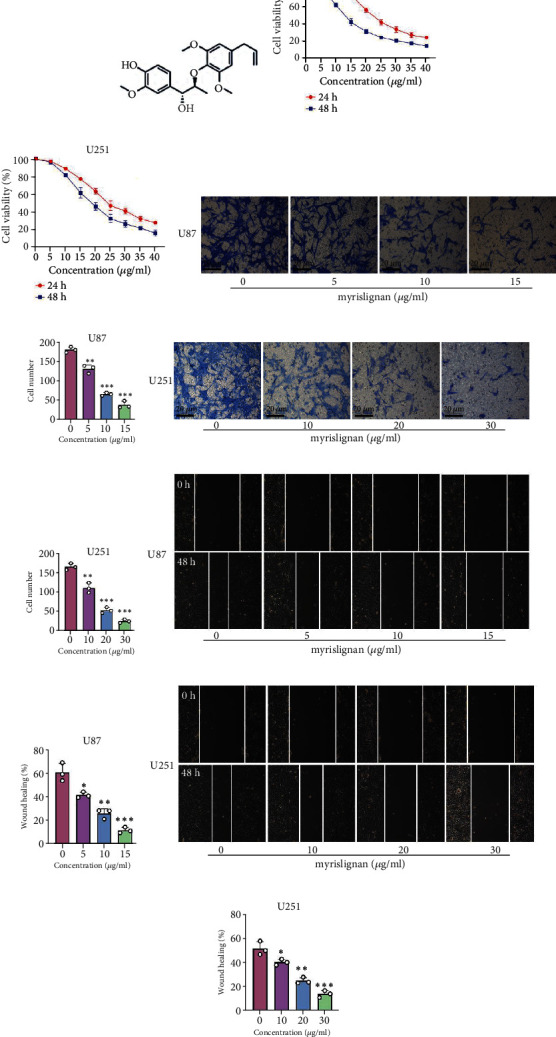
Myrislignan suppressed the growth and EMT status of GBM cells. (a) The molecular structure of myrislignan. (b, c) The cell viability of U87 and U251 was detected after treatment with increasing concentrations of myrislignan. (d–g) Transwell assay was performed to assess the migration ability of U87 and U251 cells after myrislignan treatment. Scale bar, 20 *μ*m. (h–k) Wound-healing assay was performed in U87 and U251 cells treated with myrislignan at different concentrations. Results were obtained in three independent experiments and quantified. ^∗^*P* < 0.05; ^∗∗^*P* < 0.01; ^∗∗∗^*P* < 0.001.

The chemical structure of myrislignan is shown in Figure 1(a). First, we carried out cell viability assays using CCK8 to determine the effect of myrislignan on the growth of GBM cells (U87 and U251) and to identify the appropriate concentration of myrislignan to use in subsequent assays. Myrislignan significantly inhibited the growth of U87 and U251 in a time and dosage dependent manner (Figures 1(b) and 1(c)). Based on these results, we chose specific concentrations of myrislignan to be used in subsequent analysis: 0, 5, 10, and 15 *μ*g/mL for U87 and 0, 10, 20, and 30 *μ*g/mL for U251. We then evaluated the effect of myrislignan on the EMT status of U87 and U251 cells using Transwell and wound-healing assays. We observed that myrislignan significantly inhibited the migration and invasion ability of U87 and U251 cells in a dose-dependent manner (Figures 1(d)–1(k)). These results indicated that myrislignan suppressed the growth and inhibited EMT of U87 and U251 cells *in vitro*. Consistently, the use of myrislignan also inhibited the growth of low-grade glioma cell lines SHG44 (derived from human astrocytoma), HS683 (derived from human oligodendrocyte), and SW1088 (derived from human astrocytoma) (Supplemental Figure 1A-1C).

Additionally, the general toxic effect of myrislignan was assessed in human normal astrocyte cell line NHA. The results showed that 30 *μ*g/mL (concentration with visible antiglioma effect) of myrislignan did not affected the level of apoptosis or ferroptosis associated proteins. However, higher concentration (40, 50, and 60 *μ*g/mL) of myrislignan inhibited the growth and caused lipid peroxidation of NHA cells (Supplemental Figure 1D-1F). These results indicated that low-dose myrislignan was specific for tumor inhibition.

### 3.2. Myrislignan Inhibited the Activation of NF-*κ*B Signaling Pathway and Regulated the Expression of EMT-Related Genes in GBM Cells

**Figure 2:**
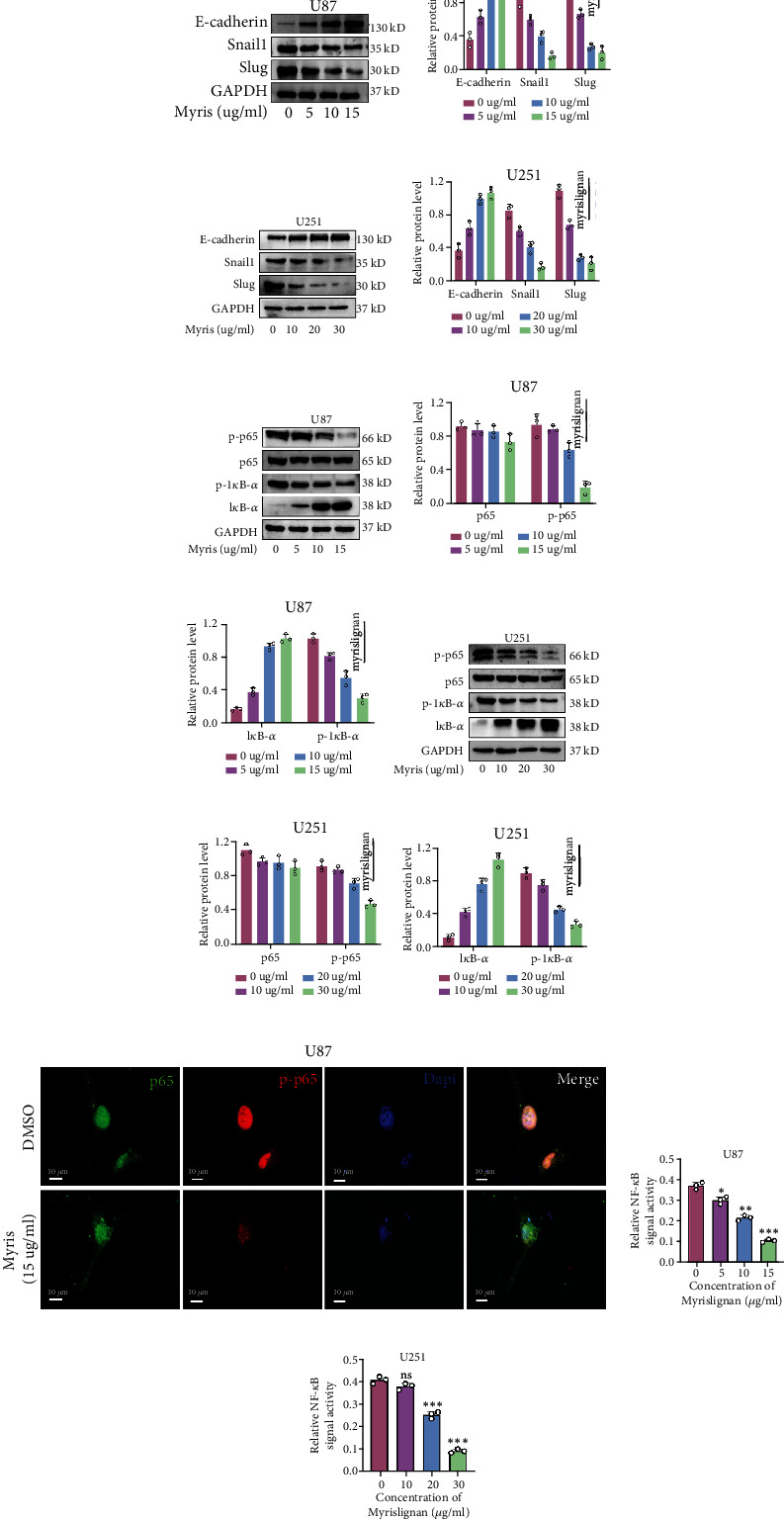
Myrislignan regulated the expression of EMT-related genes and inhibited the phosphorylation of p65 protein. (a–d) The protein (E-cadherin, Snail1, and Slug) expression was detected via western blot assay after myrislignan treatment in U87 and U251 cells. (e–j) The protein (total p65/I*κ*B-*α* and phosphorylated p65/I*κ*B-*α*) levels were measured via western blot assay after myrislignan treatment in U87 and U251 cells. (k) Immunofluorescence of p65 and p-p65 protein after myrislignan treatment in U87 cells. (l, m) Dual-luciferase reporter assay was conducted to evaluate the status of NF-*κ*B signal in GBM cells treated with myrislignan. Scale bar, 10 *μ*m. ^∗^*P* < 0.05; ^∗∗^*P* < 0.01; ^∗∗∗^*P* < 0.001; ns: no significance.

To confirm the effect of myrislignan on EMT of GBM cells, we analyzed the protein levels of some EMT-related genes including E-cadherin, Snail1, and Slug. The protein levels of Snail1, Slug, ZEB1, and Vimentin gradually decreased with increase in myrislignan concentration (Figures 2(a)–2(d) and Supplemental Figure 2). On the contrary, myrislignan treatment upregulated the expression of E-cadherin (Figures 2(a)–2(d)). However, myrislignan did not affect other EMT-associated signals such as Twist and *β*-catenin (Supplemental Figure 2).

Since myrislignan has been reported to affect the NF-*κ*B signaling pathway, we investigated if myrislignan inhibits growth and EMT of GBM cells by regulating the NF-*κ*B signaling pathway. We analyze the protein levels of total p65 and phosphorylated p65 (p-p65) using western blot and immunofluorescence and found that myrislignan decreased the levels of phosphorylated p65 protein in a dose-dependent manner, while total p65 protein levels remained unchanged (Figures 2(e)–2(h)). IkappaB-alpha (I*κ*B-*α*) is a key protein that regulates NF-*κ*B signaling. The phosphorylation of I*κ*B-*α* promotes the disaggregation of NF-*κ*B and activates the NF-*κ*B signaling pathway. In our study, myrislignan treatment downregulated phosphorylated I*κ*B-*α* levels in U87 and U251, while total I*κ*B-*α* protein levels remained unchanged. Consistent with these results, immunofluorescence imaging revealed that myrislignan treatment decreased p-p65 levels (especially nucleus p-p65) in U87 cells with no effect on the total p65 (Figure 2(i)). The dual-luciferase reporter assay was then used to determine the effect of myrislignan on NF-*κ*B signaling pathway. Myrislignan suppressed the activation of NF-*κ*B signaling in U87 and U251 cells, suggesting that myrislignan may suppress the NF-*κ*B signaling pathway by inhibiting the phosphorylation of I*κ*B-*α* and p65 protein.

### 3.3. Myrislignan Induced the Ferroptosis of GBM Cells in a System x_c_^−^-Dependent Manner

**Figure 3:**
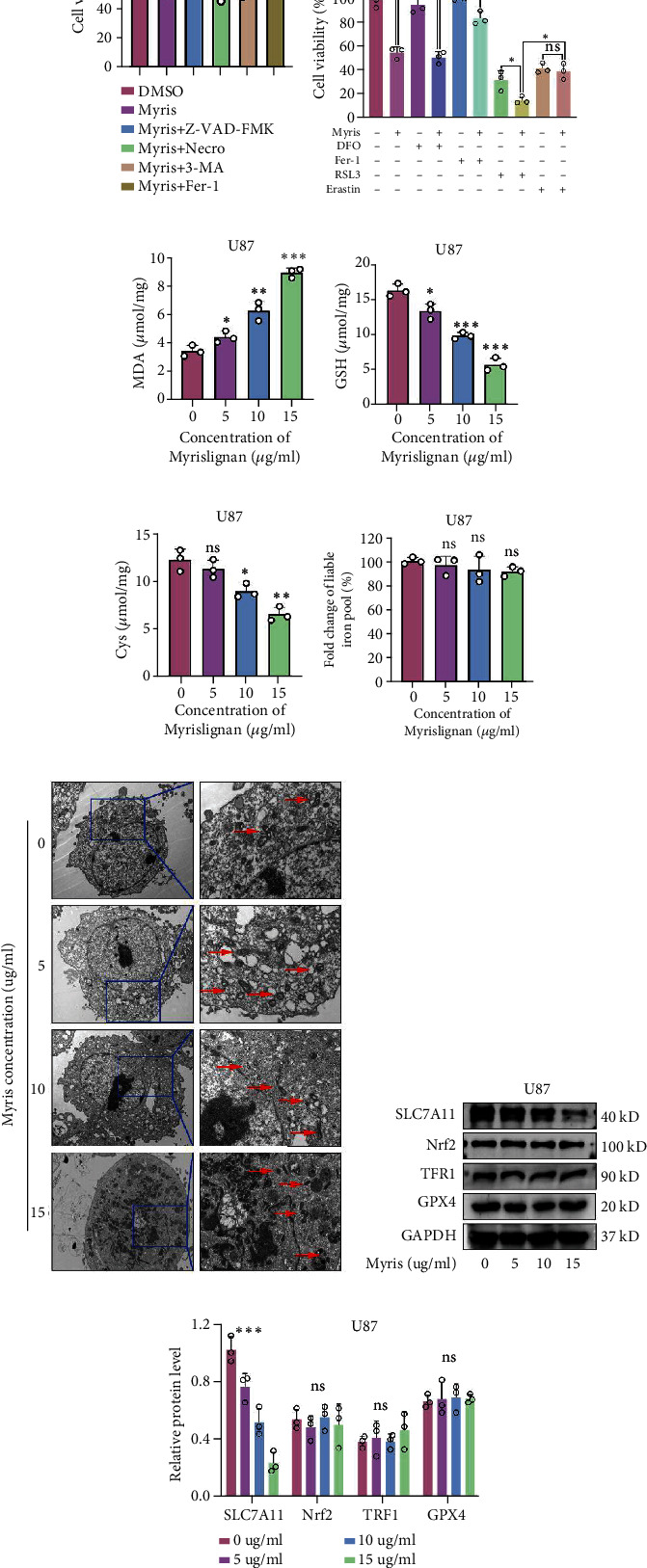
Myrislignan induced the ferroptosis of GBM cells in a system x_c_^−^-dependent manner. (a) Cell viability assay was conducted when U87 cells were treated with myrislignan (10 *μ*g/ml), Z-VAD-FMK (20 *μ*M), Necro (20 *μ*M), 3-MA (5 mM), and Fer-1 (10 *μ*M). (b) Cell viability assay was conducted when U87 cells were treated with myrislignan (10 *μ*g/ml), DFO (10 *μ*M), Fer-1 (10 *μ*M), RSL3 (2 *μ*M), and erastin (10 *μ*M). (c–f) The MDA assay, glutathione assay, Cys assay, and liable iron pool assay were conducted to measure the levels of MDA, GSH, Cys, and liable iron in U87 cells treated with increasing concentrations of myrislignan, respectively. (g) U87 cells were cultured in DMED containing increasing concentrations of myrislignan and prepared for transmission electron microscopy observation. (h, i) Protein (SLC7A11, Nrf2, TFR1, and GPX4) levels were detected after treatment with increasing concentrations of myrislignan in U87 cells. ^∗^*P* < 0.05; ^∗∗^*P* < 0.01; ^∗∗∗^*P* < 0.001; ns: no significance.

To determine the potential mechanisms of myrislignan-induced suppression of GBM proliferation, GBM cells were treated with myrislignan in combination with several classic inhibitors of cell death. The aim was to determine if any of the inhibitors reversed the myrislignan-induced suppression of growth. The results of cell viability assays in U87 showed that Z-VAD-FMK (an inhibitor of apoptosis), necrosulfonamide (Necro, a necrosis inhibitor), and 3-MA (an autophagy inhibitor) did not reverse the effects of myrislignan treatment, while ferrostatin-1 (Fer-1, a ferroptosis inhibitor) reversed myrislignan-induced growth suppressive effects (Figure 3(a)). These results indicated that myrislignan has ferroptosis-promoting effect. Iron metabolism and antilipid oxidases play key roles in the regulation of ferroptosis. To further explore the specific mechanisms by which myrislignan regulates cellular ferroptosis, U87 cells were treated with myrislignan together with inhibitors and inducers of ferroptosis. Deferoxamine (DFO), an iron chelator, failed to alleviate the myrislignan-induced growth inhibitory effects in U87 cells unlike Fer-1. Meanwhile, RSL3, an inhibitor of GPX4, enhanced the inhibitory effects of myrislignan, while erastin, an inhibitor of cystine/glutamate antiporter (system x_c_^−^), had no significant enhancement effect (Figure 3(b)). Consistently, liable iron pool levels did not change with myrislignan treatment in GBM cells, indicating that iron metabolism may not be involved in myrislignan-induced ferroptosis (Figure 3(f) and Supplemental Figure 3D). Cystine (Cys) and glutathione (GSH) are key antilipoperoxidases mainly mediated by system x_c_^−^. Myrislignan inhibited the level of Cys and GSH and eventually induced lipid peroxidation in GBM cells, manifested as the accumulation of malondialdehyde, (MDA) (Figures 3(c)–3(e) and Supplemental Figure 3A-3C). Transmission electron microscopy (TEM) revealed that myrislignan treatment induced typical features of ferroptosis in U87 cells such as atrophied mitochondria with increased membrane density and reduced mitochondrial cristae (Figure 3(g)). On the protein level, myrislignan treatment caused a decrease in the expression of SLC7A11, a component of system x_c_^−^, while Nrf2, TFR1, and GPX4 levels remained unchanged (Figures 3(h) and 3(i) and Supplemental Figure 3E-3F). In general, myrislignan induced the ferroptosis in GBM cells in a system x_c_^−^-dependent manner.

### 3.4. Myrislignan Regulated the Ferroptosis of GBM Cells via Slug-SLC7A11 Pathway

**Figure 4:**
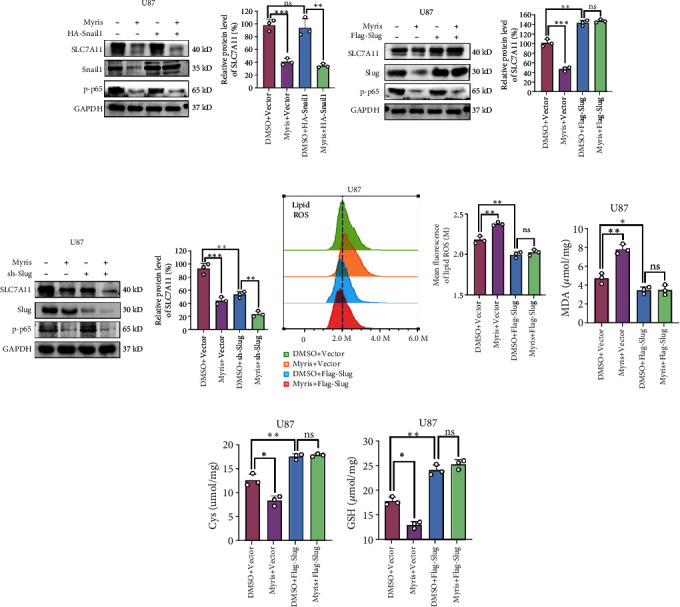
Myrislignan regulated the ferroptosis of GBM cells via Slug-SLC7A11 pathway. (a) Protein (SLC7A11, Snail1, and p-p65) levels were detected via western blot assay after myrislignan treatment with transfection of HA-Snail1 in U87 cells. (b, c) Protein (SLC7A11, Slug, and p-p65) levels were detected via western blot assay after myrislignan treatment with transfection of Flag-Slug or sh-Slug in U87 cells. (d–g) The lipid ROS assay, MDA assay, glutathione assay, and Cys assay were conducted to measure the levels of lipid ROS, MDA, GSH, and Cys in U87 cells after myrislignan treatment with transfection of Flag-Slug, respectively. ^∗^*P* < 0.05; ^∗∗^*P* < 0.01; ^∗∗∗^*P* < 0.001; ns: no significance.

We then investigated the specific mechanism of myrislignan-induced regulation of system x_c_^−^ and SLC7A11. Previous studies have proposed the potential regulation of ferroptosis by EMT-related molecules including Snail1 and Slug. In our study, we also observed that myrislignan induced a slight change in the expression of Snail1 and Slug. We then used HA-Snail1 and Flag-Slug plasmids to determine the effect of different expression levels of Snail1 and Slug on the levels of SLC7A11 and the function of system x_c_^−^. Results of western blot assays revealed that Flag-Slug significantly reversed the protein levels of SLC7A11 when myrislignan was used in GBM cells, while HA-Snail1 had no effect (Figures 4(a) and 4(b) and Supplemental Figure 4A). Moreover, knockdown of Slug enhanced the regulation of SLC7A11 level by myrislignan (Figure 4(c)). Meanwhile, overexpression of Slug reversed the effects of myrislignan on lipid ROS, MDA, Cys, and GSH in GBM cells (Figures 4(d)–4(g) and Supplemental Figure 4B-4D). These findings suggest that myrislignan regulated the ferroptosis of GBM cells via Slug-SLC7A11 pathway.

### 3.5. Myrislignan Inhibited the EMT and Suppressed the Progression of GBM *In Vivo*

**Figure 5:**
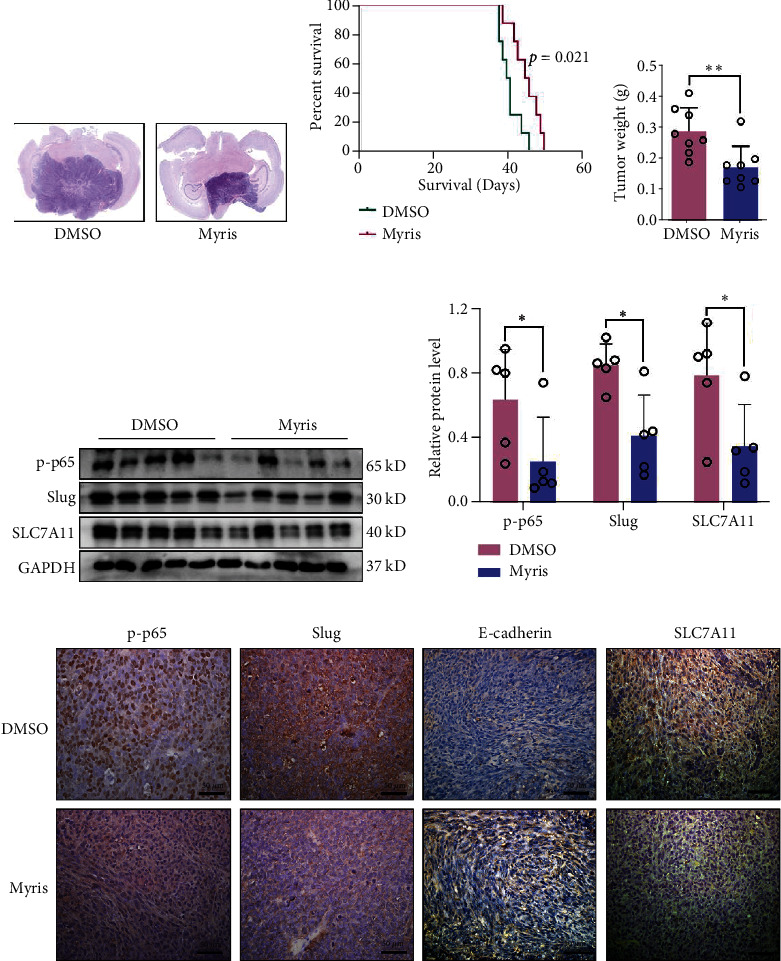
Myrislignan suppressed the progression of GBM *in vivo*. (a) Representative HE staining of mice brain sections indicated that myrislignan suppressed the growth of GBM. (b) Kaplan-Meier curves of mice survival. (c) The tumor-bearing brains were weighed. Relative weight of tumors in brain was estimated (DMSO/Myris groups—PBS groups). (d, e) Protein levels of p-p65, Slug, and SLC7A11 in mouse tumor were detected by western blot and quantified. The protein samples were extracted from tumor tissues in DMSO and Myris group of intracranial xenograft models. (f) Expression of p-p65, Slug, E-cadherin, and SLC7A11 was evaluated via IHC staining. Scale bar, 50 *μ*m. ^∗^*P* < 0.05; ^∗∗^*P* < 0.01; ^∗∗∗^*P* < 0.001.

To confirm the effect of myrislignan on GBM *in vivo*, intracranial xenograft model was constructed and treated with myrislignan. Survival analysis showed that nude mice in myrislignan-treated group had a better prognosis compared with control group. Consistently, the volume of tumor was observed visually in HE (Figure 5(a)), and the weight of glioma was calculated (Figure 5(c)). We also analyzed the protein levels of p-p65, Slug, E-cadherin, and SLC7A11 *in vivo* using western blot assay and IHC staining. Results showed that the expression of p-p65, Slug, and SLC7A11 declined in tumors of myrislignan-treated group, while the E-cadherin levels were upregulated. These results demonstrated that myrislignan inhibited EMT and suppressed the progression of GBM *in vivo*.

To research whether the cell inhibition effect of myrislignan is tumor specific *in vivo*, we simulated the administration mode of myrislignan in nude mice without tumor inoculation. Immunofluorescence of normal brain tissue showed that application of 5 mg/kg myrislignan which possessed anti-GBM activity did not induce the production of reactive oxygen species (ROS) in brain tissues, while higher concentration (20 mg/kg) of myrislignan could cause oxidative stress (Supplemental Figure 1G). This indicated that myrislignan of tumor inhibition dose had no significant inhibitory effect on normal brain tissue.

### 3.6. Myrislignan Enhanced the Ferroptosis-Promoting and Anti-GBM Activity of RSL3 *In Vitro*

**Figure 6:**
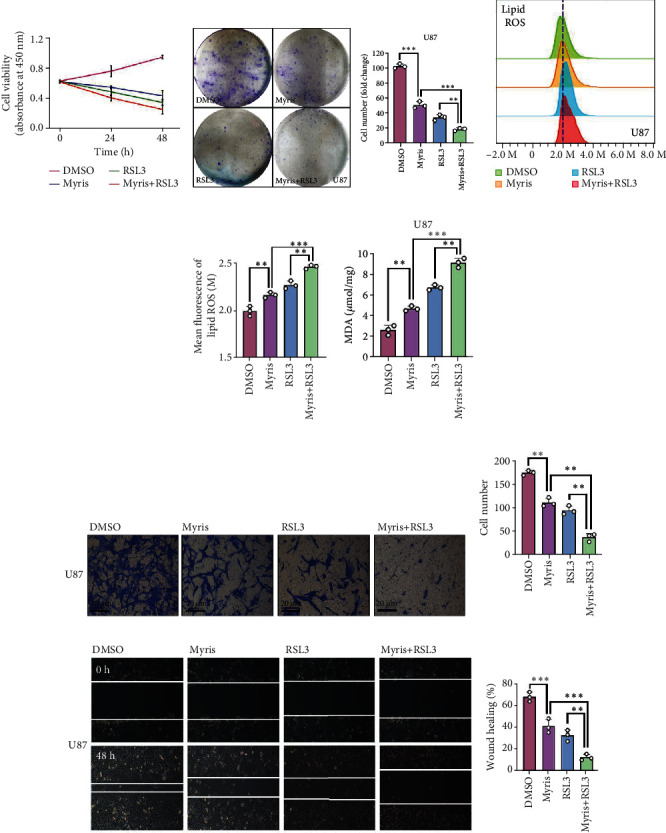
Myrislignan enhanced the ferroptosis-promoting and anti-GBM activity of RSL3 *in vitro.* (a, b) Cell viability assay and colony formation assay were conducted to evaluate the growth of U87 after treated with myrislignan and RSL3. (c, d) Lipid ROS assay and MDA assay were conducted to assess the ferroptosis-promoting effect of the treatment with myrislignan and RSL3 in U87 cells. (e–h) Wound-healing assay and Transwell assay were performed to investigate the EMT status of U87 cells after treated with myrislignan and RSL3. ^∗∗^*P* < 0.01; ^∗∗∗^*P* < 0.001.

In Figure 3(b), we mentioned that RSL3 enhanced the inhibitory effect of myrislignan in U87. We carried out cell viability and colony formation assays to evaluate the effect of myrislignan treatment combined with RSL3 on GBM cells (Figures 6(a) and 6(b) and Supplemental Figure 5A). We found that RSL3 combined with myrislignan had the strongest cytostatic effect. Consistently, on investigating the ferroptosis indicators such as lipid ROS and MDA, we found that myrislignan enhanced the ferroptosis-promoting activity of RSL3 in GBM cells (Figures 6(c) and 6(d) and Supplemental Figure 5B). Furthermore, we explored the EMT status of the cells under RSL3 and myrislignan cotreatment. Results of Transwell and wound-healing assays indicated that RSL3 promoted the anti-EMT effect of myrislignan in GBM cells (Figures 6(e)–6(h) and Supplemental Figure 5C-5F). Thus, myrislignan enhanced the ferroptosis-promoting of RSL3 and myrislignan combined with RSL3 had the strongest anti-GBM activity in GBM cells.

## 4. Discussion

GBM is the most malignant form of primary brain tumor with few effective treatments [2]. Although maximal surgical resection followed by radiotherapy and adjuvant chemotherapy has been used for treatment, the median survival for GBM patients is still very low [15]. Therefore, there is need to identify potential target genes and develop effective targeted drugs to improve the outcomes of glioma patients.

Myrislignan is a lignan isolated from *Myristica fragrans* Houtt with diverse pharmacological activities [13]. It can be metabolized by liver CYP450 enzymes and penetrate the blood-brain barrier through passive diffusion [16, 17]. It also inhibits nitric oxide production [18] and microsomes biotransformation [19]. Meanwhile, myrislignan inhibits LPS-induced inflammation in RAW 264.7 cells through the NF-*κ*B pathway [14]. NF-*κ*B is a key nuclear transcription factor that has been reported to participate in the regulation of GBM cell proliferation, migration, and apoptosis [20–22]. Hence, harnessing the pharmacology of myrislignan to treat GBM is theoretically promising.

In our study, we revealed that myrislignan suppressed NF-*κ*B signaling and affected EMT status of GBM cells. Cell viability assays revealed that myrislignan inhibited the growth of U87 and U251 cells in a time and dose-dependent manner. Consistently, when choosing specific concentrations of myrislignan to treat cells (0, 5, 10, and 15 *μ*g/mL for U87 and 0, 10, 20, and 30 *μ*g/mL for U251), we observed that myrislignan inhibited EMT in GMB cells in a dose-dependent manner. We also observed that myrislignan significantly inhibited the expression of EMT-associated genes and the phosphorylation of NF-*κ*B p65 protein. The phosphorylation of NF-*κ*B p65 is an important hallmark in the activation of NF-*κ*B signaling pathway [23] and is sufficient for the induction of EMT [24, 25]. IkappaB-alpha (I*κ*B-*α*) is also a key protein in the regulation of NF-*κ*B signaling. I*κ*B-*α* inhibits the activity of dimeric NF-kappa-B/REL complexes by trapping REL dimers in the cytoplasm through masking of their nuclear localization signals [26]. Phosphorylation of I*κ*B-*α* promotes the disaggregation of NF-*κ*B and activates the NF-*κ*B signaling pathway [27]. In our study, myrislignan significantly suppressed the phosphorylation of I*κ*B-*α* in GBM cells. Moreover, results of dual-luciferase reporter assays also revealed that myrislignan inhibited the activation of NF-*κ*B signaling pathway in GBM cells. Hence, myrislignan may inhibit NF-*κ*B signaling pathway by blocking the phosphorylation of I*κ*B-*α* and p65 which suppressed EMT of GBM cells.

Notably, we observed that myrislignan induces ferroptosis by regulating the system xc− in GBM cells. We also observed that ferrostatin-1, a ferroptosis inhibitor, partially rescued GBM cells treated with myrislignan, while inhibitors of apoptosis, necrosis, and autophagy did not reverse the effects of myrislignan treatment. These results demonstrated the ferroptosis-promoting effect of myrislignan. Ferroptosis, a unique cell death driven by iron-dependent phospholipid peroxidation, is regulated by multiple cellular metabolic events, including redox homeostasis, iron handling, and lipid metabolism [28]. The classical ferroptosis signal is system x_c_^−^-cystine/glutamate antiporter and GPX4, regulated by erastin and RSL3, respectively. The metabolism of polyunsaturated fatty acid (PUFA) is closely associated with the susceptibility of cells to ferroptosis and is mainly regulated by ACSL4 [29]. Nrf2 and TFR1 also play critical roles in accommodating lipid peroxidation and ferroptosis [30, 31]. In our study, we found that myrislignan treatment in U87 cells induced downregulation of SLC7A11, a component of system x_c_^−^ [32], while Nrf2, TFR1, and GPX4 remained unchanged. We also found that myrislignan-induced alteration of lipid peroxidation, as determined by assaying for MDA, GSH, and Cys, in GBM cells was dependent on system x_c_^−^. Labile iron pool (LIP) is mainly composed of Fe^2+^ and is an important source of intracellular oxidative radicals [33]. However, myrislignan did not affect the iron metabolism based on results from LIP assays. Altogether, myrislignan induced ferroptosis in GBM cells by regulating SLC7A11 levels and system x_c_^−^ function.

Interestingly, we found that myrislignan regulated ferroptosis by regulating EMT in GBM cells. In this part, we focused on the regulation of SLC7A11 by the Snail family. The Snail family of zinc-finger transcription factors consist of Snail1 (Snail), Snail2 (Slug), and Snail3 (Smuc), which participate in the regulation of multiple physiological processes including EMT [34]. Recently, the role of Snail family in ferroptosis has been revealed. Snail1 drove the reprogramming of EMT genes and affected ferroptosis in response to TGF-*β* [35]. Meanwhile, Snail2 regulates ferroptosis through the regulation of SLC7A11 in ovarian cancer [36]. In our study, an increase in myrislignan concentration downregulated expression of Snail1 and Slug protein levels in GBM cells. We then altered the expression of Snail1 and Slug using HA-Snail1 and Flag-Slug plasmids, respectively. Results of western blot assays indicated that Flag-Slug significantly reversed the protein level of SLC7A11 when myrislignan was used, while HA-Snail1 had no effect. Overexpression of Slug reversed the myrislignan-induced effects on lipid ROS, MDA, Cys, and GSH. Hence, overexpression of Slug rescued the level of SLC7A11 and the function of system x_c_^−^, indicating that ferroptosis was regulated through the Slug-SLC7A11 pathway. However, SLC7A11 expression is also regulated by other transcription factors such as p53, Nrf2, and ATF4 [37–39]. In our study, we did not detect any changes in Nrf2 expression when myrislignan was used (Figure 3(h)). Thus in the future, we need to determine if myrislignan regulates SLC7A11 by affecting other transcription factors represented by p53 and ATF4.

To confirm the growth suppressive effects of myrislignan in GBM, *in vivo* experiments were performed. Previous studies demonstrated that myrislignan can be rapidly eliminated from blood following intravenous (I.V.) administration to rats [40]. Meanwhile, a sensitive and accurate high-performance liquid chromatography-ultra-violet detection (HPLC-UV) method detected an accumulation of myrislignan in liver, intestine, and brain in rats after I.V. injection [17], indicating the ability of myrislignan to penetrate the blood-brain barrier (BBB). Results of survival analysis revealed that myrislignan inhibited the progression of GBM in intracranial xenografts. Furthermore, results of western blot assay and IHC staining indicated that myrislignan regulated the protein levels of p-p65, Slug, E-cadherin, and SLC7A11. Animal experiments demonstrated that myrislignan inhibited the status of EMT and suppressed the progression of GBM *in vivo*.

The classic ferroptosis inducer erastin could directly inhibit the activity of system x_c_^−^ and reduce cellular uptake of Cys [41]. However, the auxiliary use of myrislignan did not promoted the antitumor activity of erastin in Figure 3(b). Conversely, the addition of myrislignan significantly enhanced the inhibitory effect of RSL3, a key inhibitor of GPX4 [42]. Consistently, RSL3 combined with myrislignan has the strongest ferroptosis-promoting effect in U87 cells. Furthermore, RSL3 promoted the anti-EMT effect of myrislignan as observed in Transwell and wound-healing assays *in vitro*. The crosstalk between EMT and ferroptosis exists and deserves more in-depth mechanistic studies [43]. The epigenetic reprogramming of EMT promoted ferroptosis in head and neck cancer [12]. Frizzled-7 affects EMT status and identified platinum-tolerant ovarian cancer cells susceptible to ferroptosis [44]. Our study demonstrated that an NF-*κ*B inhibitor, myrislignan, induced EMT-mediated ferroptosis through regulating Slug-SLC7A11 signaling in GBM. Moreover, a novel ferroptosis-related prognostic signature revealed the EMT status in bladder cancer [45]. Hence, in the myrislignan-induced GBM suppression, there is need to determine if there is a loop of mutual regulation between EMT and ferroptosis in future studies.

**Figure 7:**
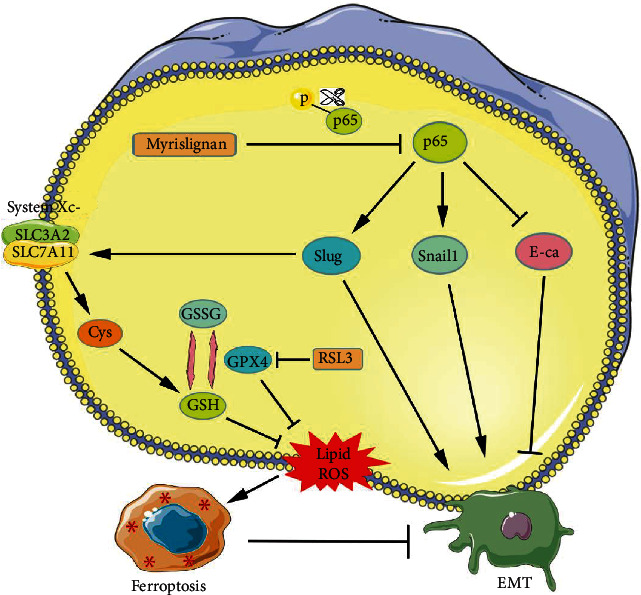
Mechanistic model for ferroptosis induced by myrislignan in GBM. Through inhibiting the phosphorylation of p65 protein, myrislignan suppressed the NF-*κ*B signaling pathway and regulated EMT-related genes including Slug, Snail1, and E-cadherin, eventually inhibiting EMT. Meanwhile, myrislignan depressed system xc − activity and induced lipid peroxidation via the Slug-SLC7A11 signaling pathway. Furthermore, myrislignan combined with RSL3 enhanced the ferroptosis via targeting SLC7A11 and GPX4, respectively, which also controlled the EMT status of GBM cells.

To conclude, our study is the first to study the effect of an NF-*κ*B inhibitor, myrislignan, in GBM. We demonstrated that myrislignan-induced ferroptosis of GBM cells by regulating EMT status in a Slug-dependent manner (Figure 7). The anti-GBM effect of myrislignan was observed *in vitro* and *in vivo*. Our findings may contribute to the development of targeted therapy against GBM and the understanding of EMT-induced ferroptosis.

## Data Availability

The data used to support the findings of this study are available from the corresponding author upon request.

## Supplementary Materials

Supplementary Materials Supplemental Figure 1: toxic effect of myrislignan in low-grade glioma and human normal astrocyte. (A-D) Cell viability assay was conducted in low-grade glioma cells (SHG44, HS683, and SW1088) and human normal astrocyte cells (NHA) treated with myrislignan. (E) The MDA assay was conducted to measure the levels of MDA in NHA cells treated with increasing concentrations of myrislignan. (F) Protein (Cyclin-D1, SLC7A11, Bcl2, and Bad) levels were detected after treatment with increasing concentrations of myrislignan in NHA cells. (G) We conducted the administration mode of myrislignan (every 3 days over a total of 21 days) with different concentrations (0, 5, 10, 15, and 20 mg/kg) in nude mice without tumor inoculation. ROS immunofluorescence of normal brain tissues was shown. ^∗^*P* < 0.05; ^∗∗^*P* < 0.01; ^∗∗∗^*P* < 0.001; ns: no significance. Supplemental Figure 2: EMT signals in U87 cells treated with myrislignan. (A-B) Protein (Twist, ZEB1, and Vimentin) levels were detected after treatment with increasing concentrations of myrislignan in U87 cells. (C-D) Phosphorylated *β*-catenin and total *β*-catenin protein levels were detected after treatment with increasing concentrations of myrislignan in U87 cells. *β*-catenin phosphorylation degree was calculated by p-*β*-catenin/total *β*-catenin. Supplemental Figure 3: myrislignan induced the ferroptosis in U251 cells. (A-D) The MDA assay, glutathione assay, Cys assay, and liable iron pool assay were conducted to measure the levels of MDA, GSH, Cys, and liable iron in U251 cells treated with increasing concentrations of myrislignan, respectively. (E-F) Protein (SLC7A11, Nrf2, TFR1, and GPX4) levels were detected after treatment with increasing concentrations of myrislignan in U251 cells. ^∗^*P* < 0.05; ^∗∗^*P* < 0.01; ^∗∗∗^*P* < 0.001; ns: no significance. Supplemental Figure 4: myrislignan regulated the ferroptosis of U251 cells in a Slug-dependent manner. (A) Protein (SLC7A11, Slug, and p-p65) levels were detected via western blot assay after myrislignan treatment with transfection of Flag-Slug in U251 cells. (B-D) The MDA assay, glutathione assay, and Cys assay were conducted to measure the levels of MDA, GSH, and Cys in U251 cells after myrislignan treatment with transfection of Flag-Slug, respectively. ^∗^*P* < 0.05; ^∗∗^*P* < 0.01; ^∗∗∗^*P* < 0.001; ns: no significance. Supplemental Figure 5: myrislignan enhanced the ferroptosis-promoting and anti-tumor activity of RSL3 in U251 cells. (A) Cell viability assay was conducted to evaluate the growth of U251 after treated with myrislignan and RSL3. (B) MDA assay was conducted to assess the ferroptosis-promoting effect of the treatment with myrislignan and RSL3 in U251 cells. (C-F) Wound-healing assay and Transwell assay were performed to investigate the EMT status of U251 cells after treated with myrislignan and RSL3. ^∗^*P* < 0.05; ^∗∗^*P* < 0.01; ^∗∗∗^*P* < 0.001.
