# Supplementary material for: NF-κB Inhibitor Myrislignan Induces Ferroptosis of Glioblastoma Cells via Regulating Epithelial-Mesenchymal Transformation in a Slug-Dependent Manner
Source: Oxid Med Cell Longev. 2023 Jan 16;2023:7098313. doi: 10.1155/2023/7098313 (PMC9870699; doi:10.1155/2023/7098313)
Supplement: Supplementary Materials — Supplemental Figure 1: toxic effect of myrislignan in low-grade glioma and human normal astrocyte. (A-D) Cell viability assay was conducted in low-grade glioma cells (SHG44, HS683, and SW1088) and human normal astrocyte cells (NHA) treated with myrislignan. (E) The MDA assay was conducted to measure the levels of MDA in NHA cells treated with increasing concentrations of myrislignan. (F) Protein (Cyclin-D1, SLC7A11, Bcl2, and Bad) levels were detected after treatment with increasing concentrations of myrislignan in NHA cells. (G) We conducted the administration mode of myrislignan (every 3 days over a total of 21 days) with different concentrations (0, 5, 10, 15, and 20 mg/kg) in nude mice without tumor inoculation. ROS immunofluorescence of normal brain tissues was shown. ∗P < 0.05; ∗∗P < 0.01; ∗∗∗P < 0.001; ns: no significance. Supplemental Figure 2: EMT signals in U87 cells treated with myrislignan. (A-B) Protein (Twist, ZEB1, and Vimentin) levels were detected after treatment with increasing concentrations of myrislignan in U87 cells. (C-D) Phosphorylated β-catenin and total β-catenin protein levels were detected after treatment with increasing concentrations of myrislignan in U87 cells. β-catenin phosphorylation degree was calculated by p-β-catenin/total β-catenin. Supplemental Figure 3: myrislignan induced the ferroptosis in U251 cells. (A-D) The MDA assay, glutathione assay, Cys assay, and liable iron pool assay were conducted to measure the levels of MDA, GSH, Cys, and liable iron in U251 cells treated with increasing concentrations of myrislignan, respectively. (E-F) Protein (SLC7A11, Nrf2, TFR1, and GPX4) levels were detected after treatment with increasing concentrations of myrislignan in U251 cells. ∗P < 0.05; ∗∗P < 0.01; ∗∗∗P < 0.001; ns: no significance. Supplemental Figure 4: myrislignan regulated the ferroptosis of U251 cells in a Slug-dependent manner. (A) Protein (SLC7A11, Slug, and p-p65) levels were detected via western blot assay after myrislign [file 7098313.f1.docx]

**Supplemental Figures and Figure Legends**


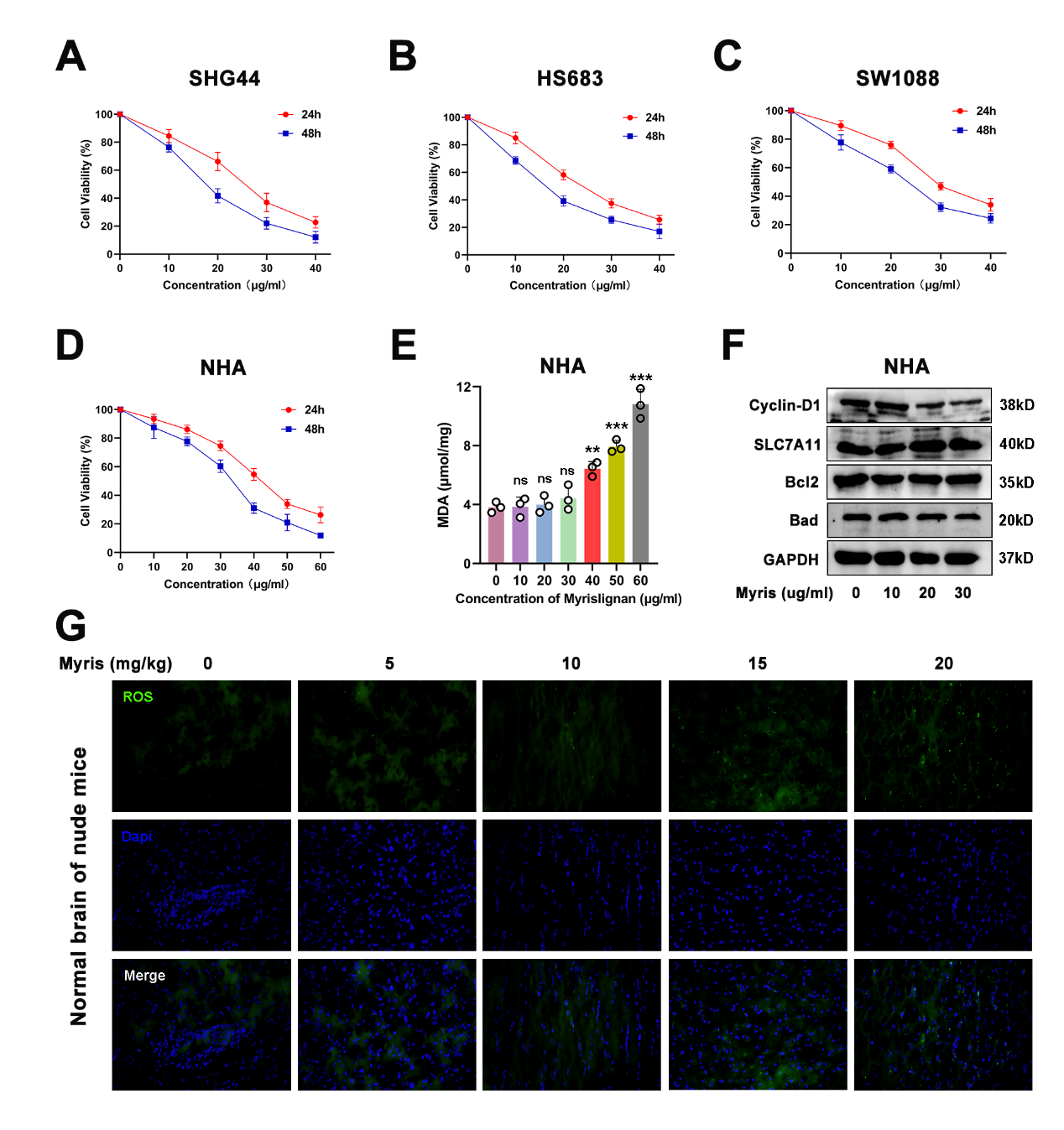


Supplemental Figure 1. Toxic effect of myrislignan in low grade glioma and human normal astrocyte.

(A-D) Cell viability assay was conducted in low grade glioma cells (SHG44, HS683 and SW1088) and human normal astrocyte cells (NHA) treated with myrislignan. (E) The MDA assay was conducted to measure the levels of MDA in NHA cells treated with increasing concentrations of myrislignan. (F) Protein (Cyclin-D1, SLC7A11, Bcl2 and Bad) levels were detected after treatment with increasing concentrations of myrislignan in NHA cells. (G) We conducted the administration mode of myrislignan (every 3 days over a total of 21 days) with different concentrations (0, 5, 10, 15 and 20mg/kg) in nude mice without tumor inoculation. ROS immunofluorescence of normal brain tissues was shown. *, P<0.05; **, P<0.01; ***, P<0.001; ns, no significance.


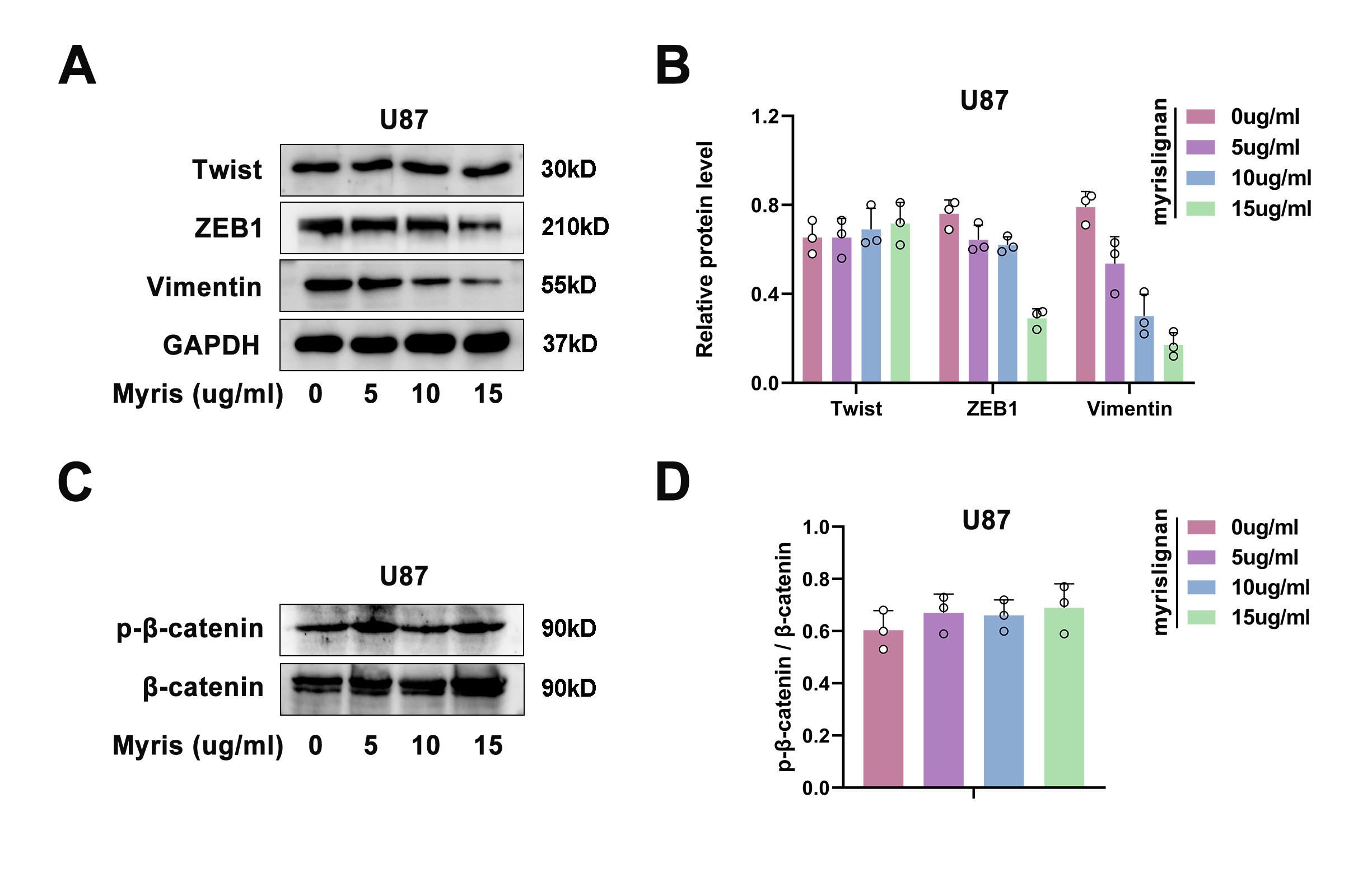


Supplemental Figure 2. EMT signals in U87 cells treated with myrislignan.

(A-B) Protein (Twist, ZEB1 and Vimentin) levels were detected after treatment with increasing concentrations of myrislignan in U87 cells. (C-D) Phosphorylated β-catenin and total β-catenin protein levels were detected after treatment with increasing concentrations of myrislignan in U87 cells. β-catenin phosphorylation degree was calculated by p-β-catenin / total β-catenin.


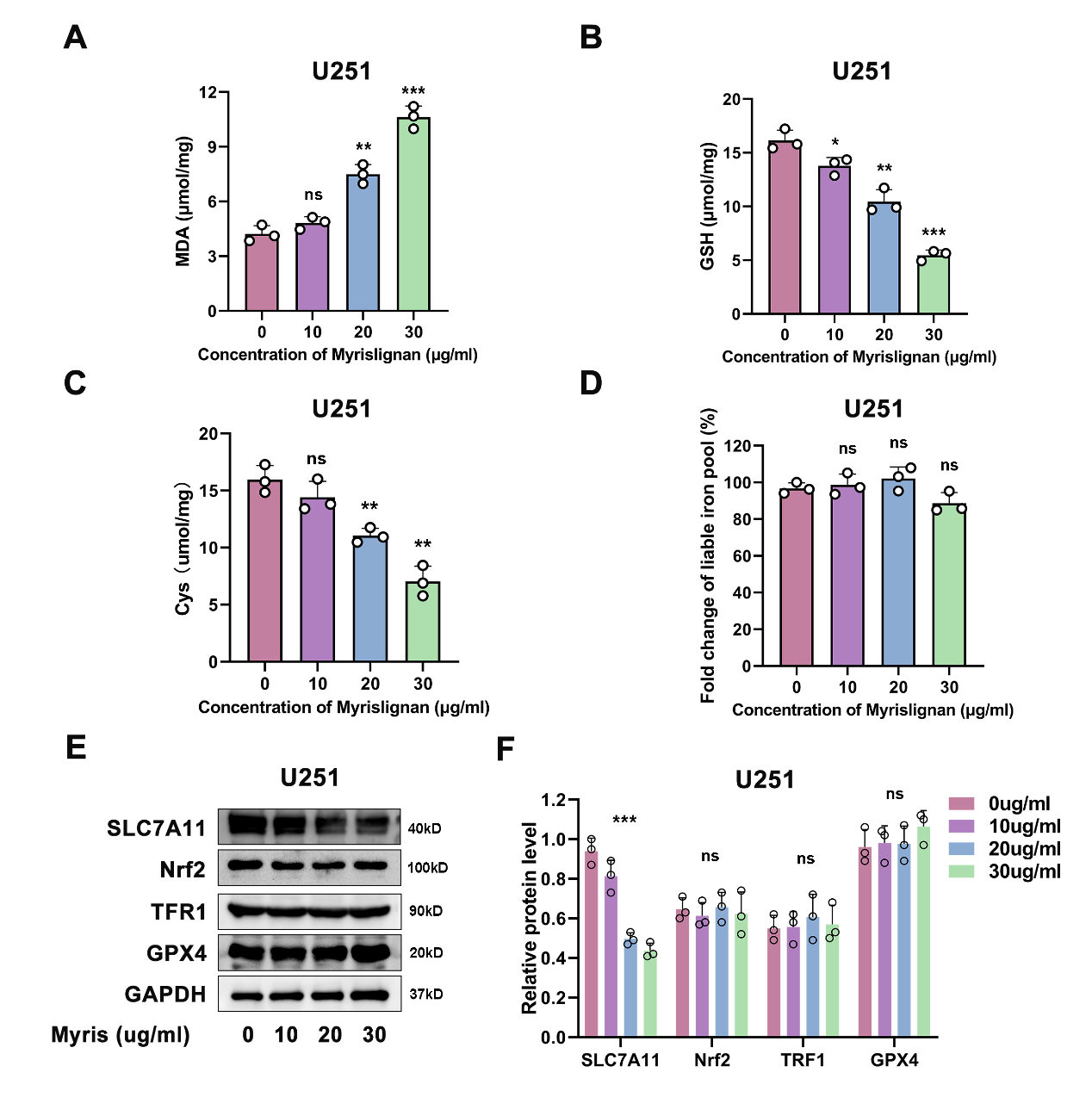


Supplemental Figure 3. Myrislignan induced the ferroptosis in U251 cells.

(A-D) The MDA assay, glutathione assay, Cys assay and liable iron pool assay were conducted to measure the levels of MDA, GSH, Cys and liable iron in U251 cells treated with increasing concentrations of myrislignan, respectively. (E-F) Protein (SLC7A11, Nrf2, TFR1 and GPX4) levels were detected after treatment with increasing concentrations of myrislignan in U251 cells. *, P<0.05; **, P<0.01; ***, P<0.001; ns, no significance.


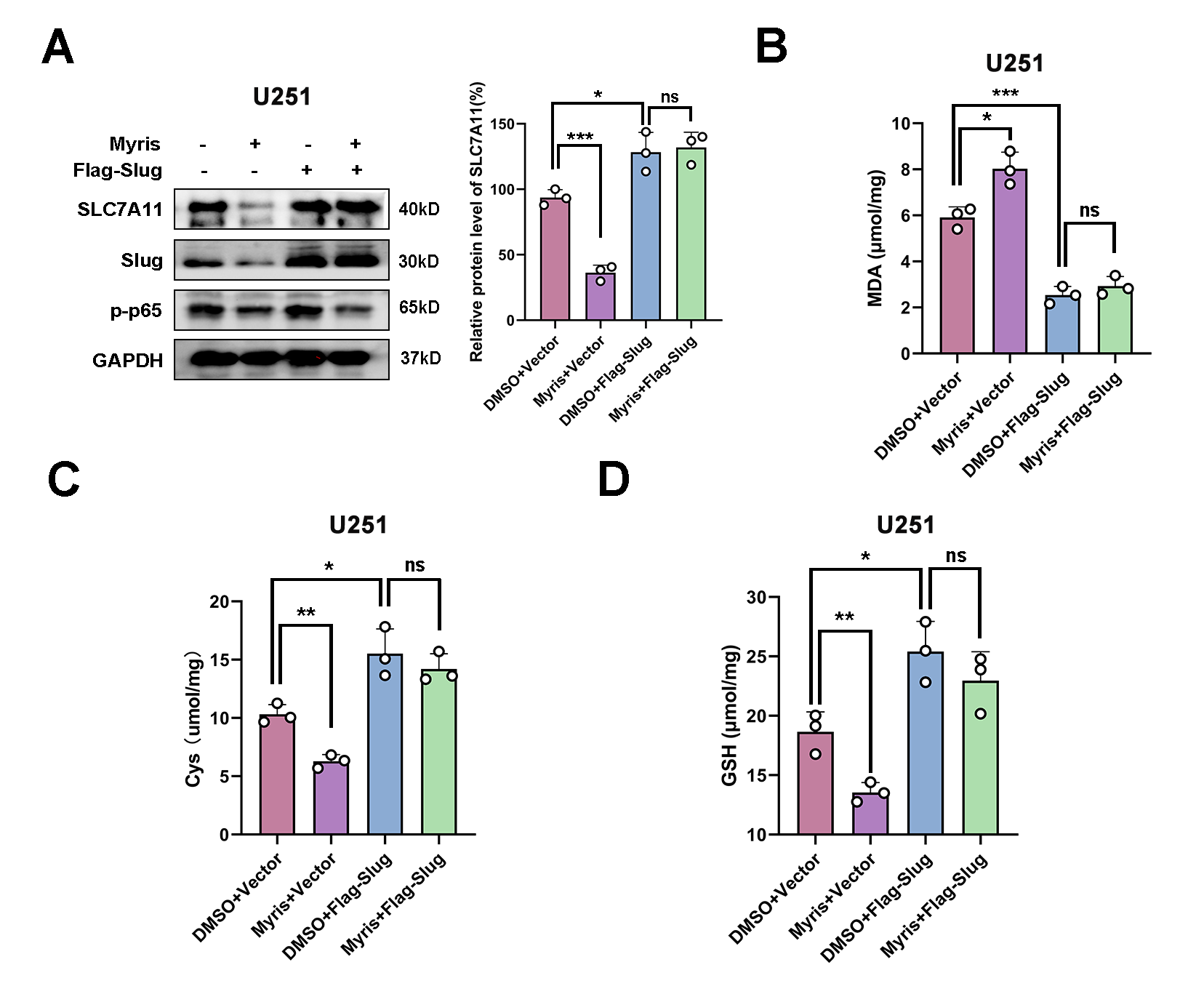


Supplemental Figure 4. Myrislignan regulated the ferroptosis of U251 cells in a Slug-dependent manner.

(A) Protein (SLC7A11, Slug and p-p65) levels were detected via western blot assay after myrislignan treatment with transfection of Flag-Slug in U251 cells. (B-D) The MDA assay, glutathione assay and Cys assay were conducted to measure the levels of MDA, GSH and Cys in U251 cells after myrislignan treatment with transfection of Flag-Slug, respectively. *, P<0.05; **, P<0.01; ***, P<0.001; ns, no significance.


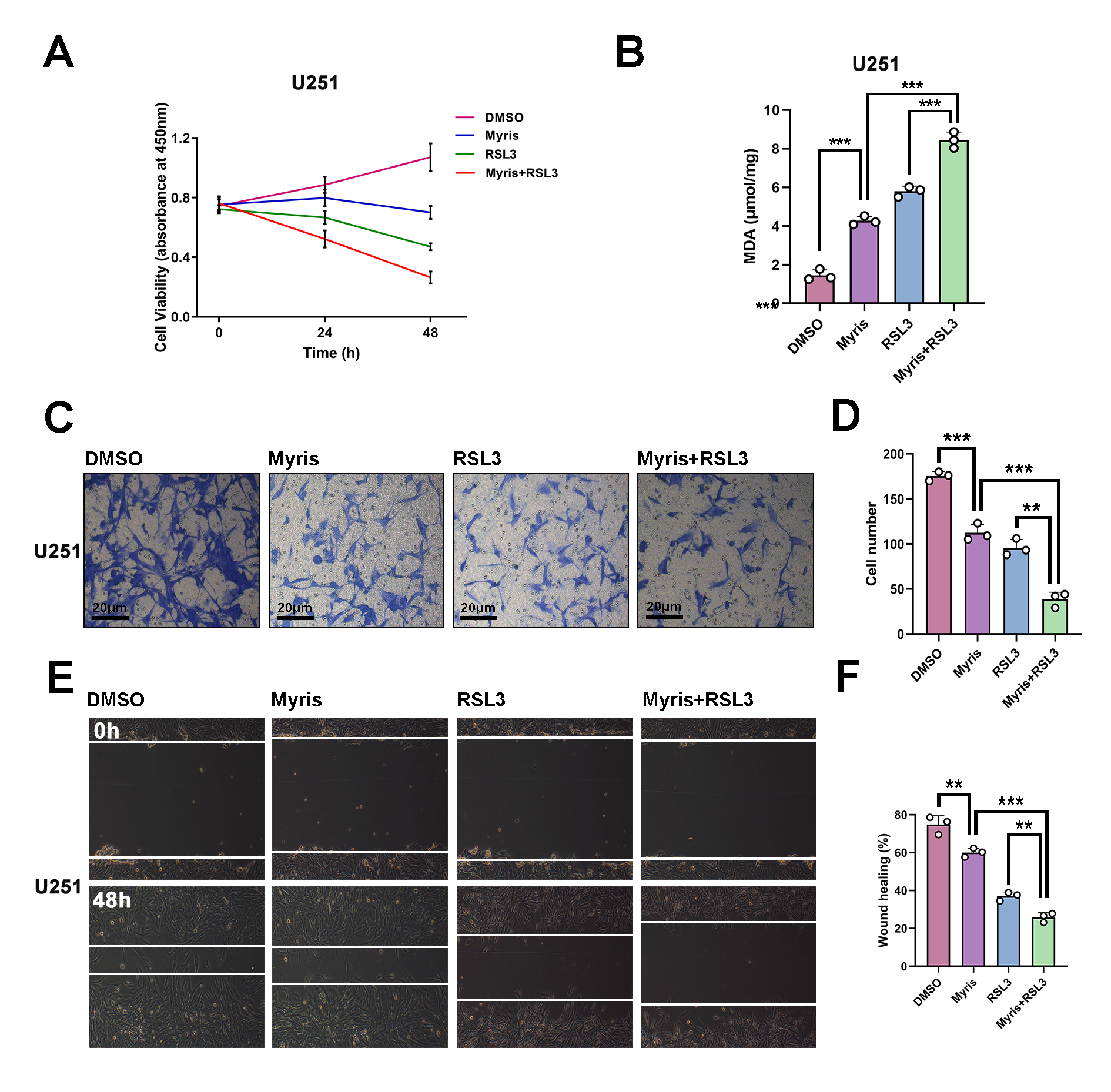


Supplemental Figure 5. Myrislignan enhanced the ferroptosis-promoting and anti-tumor activity of RSL3 in U251 cells.

(A) Cell viability assay were conducted to evaluate the growth of U251 after treated with myrislignan and RSL3. (B) MDA assay were conducted to assess the ferroptosis-promoting effect of the treatment with myrislignan and RSL3 in U251 cells. (C-F) Wound-healing assay and transwell assay were performed to investigate the EMT status of U251 cells after treated with myrislignan and RSL3. *, P<0.05; **, P<0.01; ***, P<0.001.
